# Do medical treatment choices affect the health of chronic patients in middle and old age in China?—Evidence from CHARLS 2018

**DOI:** 10.1186/s12889-022-13309-3

**Published:** 2022-05-10

**Authors:** Shaoliang Tang, Ying Gong, Ling Yao, Yun Xu, Meixian Liu, Tongling Yang, Chaoyu Ye, Yamei Bai

**Affiliations:** Nanjing University of Chinese Medicine, Nanjing, China

**Keywords:** Medical Treatment Choice, Chronic Patients in Middle and Old Age, Health Level, Integrated Chinese and Western Medicine, Disease Groups

## Abstract

Different medical treatment choices may affect the health of patients with chronic diseases. This study aims to assess the relationship between treatment choices, including the use of traditional Chinese medicine (TCM), and the health levels of middle-aged and elderly patients with six chronic diseases. The sample data comes from China Health and Retirement Longitudinal Study (CHARLS 2018). Basic conditions, medical choices and health status of patients are incorporated. The ordered Logit and Logit regression models are used to analyze and compare the effects of six chronic disease patients’ medical options on their self-rated health (SRH) and depression. The overall average score of SRH is the highest in patients with heart disease (the worst in SRH), which is 3.433. Arthritis patients have the highest overall depression average score (depression) at 0.444. Under the premise of controlling a variety of socio-demographic factors, compared with the non-treatment group, taking TCM has a significant positive effect on SRH of patients with five diseases except hypertension. Both taking western medicine (WM) and taking integrated Chinese and Western medicine (IM) have a significant positive effect on SRH scores of patients with six chronic diseases in middle and old age. Taking TCM has effect on depression of patients with heart or stomach diseases, and taking WM and IM affects depression of middle-aged and elderly chronic patients except diabetes. Taking IM has a greater effect on SRH and depression of chronically ill patients, followed by taking WM, and the effect of taking TCM is relatively small, which is related to the development stage of the disease. Therefore, in the future, the control and treatment of chronic diseases in the middle and late stages can be discussed from the perspective of integrated traditional Chinese and western medicine, but attention should be paid to drug interactions. In order to improve the treatment rate and health level of patients with chronic diseases, their economic burden should be reduced, and they should be guided to choose more reasonable treatment methods.

**Supplementary Information:**

The online version contains supplementary material available at 10.1186/s12889-022-13309-3.

## Introduction

### The burden of chronic diseases

Chronic diseases are a major threat to human health. The World Health Statistics 2020 released by the World Health Organization showed that although the mortality rate caused by chronic diseases had decreased, the total number of deaths caused by chronic diseases was increasing due to the increase in the prevalence. Compared with 2000, the total number of additional healthy life lost due to heart disease, diabetes, stroke, lung cancer and chronic obstructive pulmonary disease globally in 2019 was nearly 100 million years. After the COVID-19 outbreak, complications such as cardiovascular disease, diabetes, chronic lung disease and cancer had significantly increased the mortality rate [1]. Therefore, all countries need to strengthen the prevention, diagnosis and treatment of non-communicable diseases.

With the development of economy and society and the improvement of the level of health care, China’s overall life expectancy continued to grow, reaching 76.6 years by 2018, which was significantly higher than the world average [2]. However, the aging of the population and the acceleration of the industrialization process have increased the risk factors for the onset of chronic diseases. The health expectancy of Chinese people still accounts for a low proportion of the overall life expectancy. Coupled with the prolonged survival of patients with chronic diseases, it has become a norm for the elderly to live with diseases. Among them, hypertension [3], cardiovascular disease [4], arthritis [5], diabetes [6], stomach or digestive system diseases [7], etc. are the most common diseases, which seriously affect the health and living conditions of middle-aged and elderly people. Therefore, in-depth research on the health status of the elderly with chronic diseases is even more important. This article focuses on the top six chronic diseases with the highest prevalence in CHARLS 2018: hypertension, arthritis or rheumatism, diabetes, stomach or digestive system diseases, dyslipidemia, heart disease.

### Medical treatment choices of chronic patients

Nowadays, patients are faced with multiple choices when treating diseases, such as medicine, radiation, surgery, massage therapy and traditional Chinese medicine. In many countries, medical pluralism had become the norm, and western medicine and traditional medicine coexisted in the market [8]. The right of patients to decide on their own medical treatment originated from the development of medicine and culture in the mid-twentieth century, including the consumer movement of medical insurance, the dominance of third-party payment reimbursement schemes, and the rise of bioethics as a medical force [9]. The elderly who have access to medical information and treatment options also benefit from this social trend. Due to the longer course and slower changes in the condition, patients with chronic diseases have more medical choices and greater initiative. This article will focus on the medical choices of the middle-aged and elderly patients with chronic diseases. At the same time, Chinese patients have always been in a medical environment where Chinese and Western medicine coexist. Traditional Chinese medicine has developed for thousands of years with its complete theoretical system. In modern society, Western medicine has gradually dominated the health system. The core concept of Chinese medicine emphasizes the use of gentle methods to reshape the balance of the body, which is also advocated by most Chinese. Therefore, China is also an important environment for studying people’s medical choices [10].

Based on this, the medical choice defined in this paper is the choice of medical treatment, and mainly the choice of Chinese and Western medicine, which reflects patients’ comprehensive understanding of their own diseases and preference for treatment behavior, and plays an important role in the treatment process [11]. Based on existing research on medical choice, it mainly refers to the behavioral choice of individuals seeking medical help in the public health care system. This is inseparable from the patient’s own socio-economic background and living environment, and is a process involving the integration of the patient’s behavior and psychology.

Traditional medicine is generally regarded as part of cultural traditions. In many countries, traditional medicine was also called complementary medicine, which was represented by ingredients extracted from natural products [12]. It had the characteristics of being easy to apply, cheap and accessible, and less toxic [13], and had been proven to have beneficial effects on chronic diseases and anti-cancer [14]. Studies found that the cultural connotation of traditional medicine increased the tendency of people with specific values and beliefs to use it [15]. The increasing popularity of health care [16] and the more friendly relationship between doctors and patients [17] had become the reasons why patients chose traditional medicine. WM is evolved from the western philosophical way of thinking and is based on natural science. The development of WM is based on human body structure and anatomy, so the research of WM is also based on the way of thinking that decomposes the human body into several independent parts, which is the driving force for the development of WM [18]. Based on WM’s reductionist criteria, the holistic view of health advocated by TCM is deserving of criticism because it obscures the social roots of disease [19]. Surveys from Hong Kong showed that many people retained the “old-fashioned” concept of Chinese medicine, viewing Chinese medicine as a non-scientific practice, and had more trust in the credibility of Western medicine [20]. Portugal has a long tradition in traditional Western healthcare, while CAM and WM are considered complementary options for individuals during the diagnosis and treatment stages [21].

Regarding the impact of the medical treatment choice on the health of patients, most researches focus on verifying the effects of different treatments on patients’ clinical manifestations from the perspective of treatment effectiveness, but few studies examine the impact of their medical choices on their own health from patients’ points of view.

### Research on integrated traditional Chinese and Western medicine

The combined use of traditional medicine and conventional medicine is also common in elderly patients with chronic diseases [22]. At present, the scope of research on integrated traditional Chinese and Western medicine has gone beyond the evaluation of specific treatments, mainly including the study of nursing models for optimized treatments, the evaluation of multi-mode holistic system intervention in physical and mental health, the relationship between doctors and patients, the promotion of computer-assisted methods, and the individualization of treatment measures [23]. Some studies have shown that patients treated with IM have better therapeutic effects than patients treated with WM alone, including lowering blood pressure [24], treating rheumatoid arthritis [25], and promoting postoperative gastrointestinal function recovery [26]. Depression-related research suggested that in the early stage of depression, it was appropriate to use traditional Chinese medicine as the main treatment method with the participation of psychotherapy. In the mid-term, it must be treated with a sufficient amount of integrated Chinese and Western medicine for a full course of treatment [27]. Overall, however, systematic reviews of the efficacy of CAM treatment have not been accepted, more well-designed studies are needed for further confirmation. As the elderly often experience several chronic diseases at the same time, future research can explore how the integration of Chinese and Western medicine can help control comorbidities.

### Marginal contribution of the study

In view of the diversification of medical treatment choices, this study will choose a new perspective to explore based on the model of factors that influence health level recognized by the academic community. The study will use high-quality nationwide large sample data to focus on the association between medical treatment choices and the health of middle-aged and elderly patients with six chronic diseases in China. CHARLS is a large-scale longitudinal survey project of middle-aged and elderly people (≥ 45 years old) hosted by Peking University and approved by the Peking University Ethics Review Committee. It adopts a multi-stage, stratified, probability ratio scale sampling design, involving respondents from 28 provinces in Mainland China and provides statistical information such as medical treatment choices and health levels of chronic patients for this study. Since the use rate of TCM for patients with chronic diseases in the CHARLS database was stable between 2011 and 2018 [28], this study selected the latest public follow-up survey data in 2018 and established regression models. The marginal contribution of this research lies in the innovative combination of chronic diseases and medical treatment choices. This can better reveal the prevalence of TCM and WM in several common chronic diseases and its effect on patients’ health. In addition, in order to comprehensively assess the health of middle-aged and elderly people, this paper examines the physical health represented by self-rated health and the mental health represented by depression.

## Methods

### Study design and patients

CHARLS is a large-scale longitudinal survey project of middle-aged and elderly people (≥ 45 years old) hosted by Peking University and approved by the Peking University Ethics Review Committee. The survey aims to conduct high-quality micro-data analysis of population health, including scientific and policy research on topics such as population aging, health economics, and social security. It adopts a multi-stage, stratified, probability ratio scale sampling design, involving respondents from 150 counties and 450 villages in 28 provinces (autonomous regions and municipalities) in Mainland China. The CHARLS national baseline survey was conducted in 2011, and follow-up surveys were conducted in 2013, 2015, and 2018. This study was conducted on CHARLS 2018 respondents who were 45 years of age or older and had a physician-diagnosed condition of any of the following: hypertension, diabetes, dyslipidemia, heart disease, stomach or digestive disease, arthritis, or rheumatism.

### Variable description

As the most basic demographic characteristics, age and gender [29] are usually included in the model to study the level of health, especially when the research object is the elderly group. The study finds that public health insurance system can improve personal health [30]. Therefore, the basic impact of medical insurance is also taken into consideration. Socio-economic status (SES) [5], which is usually conceptualized as income, education or occupation, and social contact frequency [31] and drinking [32] are also related to disease incidence and health status. In addition, according to the difference of China’s geographical location and economic development, this study divides the whole country into four major plates: eastern, central, western and northeast, and investigates the differentiated health status of different regions.

Based on this, the research independent variable included in this study is medical treatment choice (taking TCM, taking WM, taking IM, other treatment methods other than taking medicine, none of the above). The dependent variable is the health level (self-rated health, depression). The control variables are a series of social demographic characteristics and health behaviors, including: age, gender, personal income (Since the income variable is a large number in absolute value, taking the logarithm of income scales the data to reduce heteroscedasticity), region, social activities, drinking, education level, and medical insurance. The variables and their measurements are shown in Table 1.

**Table 1:** Variable description

Variable type	Variable	Description of variable setting
**Independent variable**	**Medical treatment choice**	Taking Chinese traditional medicine = 1, Taking Western modern medicine = 2, Other treatments = 3, Integrated Traditional Chinese and Western Medicine = 4, None of the above = 0
**Dependent variable**	**Self-rated health**	Very good 1–2-3–4-5 Very poor
	**Depression**	Yes = 1, No = 0
**Control variable**	**Age**	45 years old and above
	**Gender**	Male = 1, Female = 2
	**Income**	Taking the logarithm
	**Region**	Eastern Region = 1, Central Region = 2, Western Region = 3, Northeast Region = 4
	**Social activities**	Yes = 1, No = 0
	**Education**	No formal education (illiterate) = 1, Did not finish elementary school = 2, Home school = 3, Elementary school = 4, Middle school = 5, High school = 6, Vocational school = 7, Two-/Three-Year College / Associate degree = 8, Four-Year College / Bachelor’s degree = 9, Post-graduate, Master’s degree = 10, Post-graduate, Doctoral degree = 11
	**Drinking**	Drink more than once a month = 1, Drink but less than once a month = 2, None of these = 3
	**Medical insurance**	Urban employee medical insurance = 1, Urban and rural resident medical insurance = 2, Urban resident medical insurance = 3, New rural cooperative medical insurance = 4, None of the above = 5

### Statistical analysis

In this study, the dependent variable SRH is an ordered multi-categorical variable with discrete values ranging from 1–5. The dependent variable depression is a binary variable with a value of 0 or 1, which violates the normality assumption of residuals, and the variance of residuals is not a fixed value, and the relationship between the independent variable and the dependent variable is non-linear.

Therefore, the ordered logit regression model and the logit regression model are used for estimation.

Establish the following measurement model:

1 $${SH}_{i}={a}_{0}+{a}_{1}{mc}_{i}+{\gamma }_{1}{X}_{1i}+{\varepsilon }_{1i}$$

2 $${Depression}_{i}={b}_{0}+{b}_{1}{mc}_{i}+{\gamma }_{2}{X}_{2i}+{\varepsilon }_{2i}$$

Among them, in formula (1), ${SH}_{i}$ is the self-rated health score of the i-th chronic disease patient; ${a}_{0}$ is the intercept term; ${mc}_{i}$ stands for medical choice. In order to prevent the occurrence of “dummy variable trap”, taking non-treatment as the benchmark variable, here we introduce several dummy variables of taking TCM, taking WM, other treatments, taking IM and taking insulin injection. ${X}_{1i}$ is a set of control variables; ${\varepsilon }_{1i}$ is a disturbance term. In formula (2), ${Depression}_{i}$ is the depression score of the i-th chronic disease patient; ${b}_{0}$ is the intercept item; ${mc}_{i}$ represents the medical choice; ${X}_{2i}$ is a set of control variables; ${\varepsilon }_{2i}$ is the disturbance item.

The follow-up will focus on analyzing the size, direction and significance level of the dummy variable coefficients contained in ${mc}_{i}$ and the average marginal effect (AME) of all samples.

## Results

### Characteristics of patients with six chronic diseases

As shown in Table 2, the average age of patients with the six diseases is over 60 years old. Among them, the average age of heart disease patients is the oldest, and the average age of gastric patients is the youngest. In terms of gender, the proportion of female patients with the six diseases is higher than that of males. From the perspective of social activities, arthritis patients have the lowest participation in social activities, and the proportion of people with other chronic diseases participating in social activities is more than 50%. Among the education levels, the majority of chronically ill patients have junior high school education or below. Among the personal incomes, after taking the logarithm, the average personal income of diabetic patients is higher, and the average personal income of chronic disease patients suffering from arthritis is relatively low. Among all the samples of chronic disease patients, the number of people living in the western region is the largest. In addition, only no more than a quarter of patients drink alcohol every month, with the lowest proportion of patients with heart disease. Less than 5% of patients with chronic diseases do not have basic medical insurance, and the proportion of patients with new rural cooperative medical insurance ranges from 56 to 68%.

**Table 2:** Basic characteristics of chronic patients with six diseases

Variable	Hypertension	Arthritis	Diabetes	Stomach disease	Dyslipidemia	Heart disease	*P*-value
	(Mean/Proportion%)						
**Age**	64.27	63.52	63.75	62.26	62.75	64.98	< 0.001
**Gender**							
male	47.37	40.03	43.97	42.19	45.44	38.66	< 0.001
female	52.63	59.97	56.03	57.81	54.56	61.34	
**Ln (Income)**	6.28	5.79	6.61	5.84	6.60	6.35	< 0.05
**Region**							
east	31.79	23.86	34.18	25.94	30.57	27.06	< 0.001
midland	28.21	28.74	30.46	29.85	30.97	26.54	
west	32.82	41.46	28.14	38.49	30.30	33.65	
northeast	7.18	5.94	7.22	5.72	8.16	12.75	
**Social activities**							
no	47.17	50.69	44.84	47.84	40.96	47.15	< 0.001
yes	52.83	49.31	55.16	52.16	59.04	52.85	
**Education**							
No formal education (illiterate)	24.16	28.58	22.78	25.30	18.80	24.14	< 0.001
Did not finish primary school	21.15	23.71	19.75	22.47	18.16	20.22	
Elementary school	22.27	21.53	21.23	23.18	21.06	22.13	
Middle school	20.06	18.22	20.93	18.66	23.32	19.43	
High school and above	12.36	7.97	15.32	10.39	18.65	14.09	
**Drinking**							
Drink more than once a month	24.97	22.97	20.75	22.36	23.57	18.77	< 0.001
Drink but less than once a month	6.72	6.73	7.06	7.44	7.97	7.09	
None of these	68.32	70.30	72.19	70.20	68.45	74.14	
**Medical Insurance**							
Urban employee medical insurance	15.12	14.17	20.99	11.79	22.95	18.71	< 0.001
Urban and rural resident medical insurance	13.33	12.12	12.58	11.84	10.68	10.37	
Urban resident medical insurance	4.47	4.24	5.74	3.77	5.81	5.73	
New rural cooperative medical insurance	62.09	64.48	56.04	68.08	55.65	60.37	
None of the above	4.98	4.99	4.65	4.51	4.92	4.83	

### Medical treatment choice and health status of patients with six chronic diseases

Table 3 shows the specific conditions of medical choices for patients with six diseases in 2018. The prevalence of WM use in hypertensive patients is the highest among the six diseases, which is 63.75% (95%CI 62.59% to 64.90%). Arthritis patients who choose to take other treatment methods other than medication are the most relative to other diseases, with prevalence rate of 24.58% (95%CI 23.60% to 25.59%). The prevalence of TCM use for diabetic patients is the lowest among the six diseases, which is 3.80% (95%CI 3.10% to 4.65%). The prevalence of heart disease patients taking TCM and IM is the highest among the six diseases, with 10.03% (95%CI 9.10% to 11.04%) and 13.35% (95%CI 12.29% to 14.49%), respectively.

**Table 3:** Medical choices for patients with six chronic diseases

Medical Treatment Choice	Disease groups(95%CI), %						*P*-value
	**Hypertension**	**Arthritis**	**Diabetes**	**Stomach disease**	**Dyslipidemia**	**Heart disease**	
None	20.27 (19.32 to 21.25)	35.56 (34.46 to 36.67)	26.62 (24.88 to 28.44)	35.53 (34.30 to 36.79)	34.50 (33.06 to 35.98)	28.09 (26.66 to 29.57)	< 0.001
Taking Chinese traditional medicine	4.01 (3.56 to 4.51)	6.87 (6.31 to 7.48)	3.80 (3.10 to 4.65)	8.06 (7.37 to 8.80)	4.55 (3.95 to 5.23)	10.03 (9.10 to 11.04)	
Taking Western modern medicine	63.75 (62.59 to 64.90)	20.72 (19.80 to 21.67)	55.99 (53.98 to 57.98)	38.31 (37.05 to 39.59)	40.35 (38.86 to 41.87)	41.42 (39.83 to 43.02)	
Other treatments	6.22 (5.67 to 6.83)	24.58 (23.60 to 25.59)	6.88 (5.93 to 7.97)	5.88 (5.29 to 6.52)	10.79 (9.87 to 11.78)	7.11 (6.32 to 7.99)	
Integrated Traditional Chinese and Western Medicine	5.74 (5.21 to 6.33)	12.27 (11.53 to 13.05)	6.71 (5.77 to 7.79)	12.22 (11.39 to 13.10)	9.81 (8.93 to 10.76)	13.35 (12.29 to 14.49)	

**Fig. 1:**
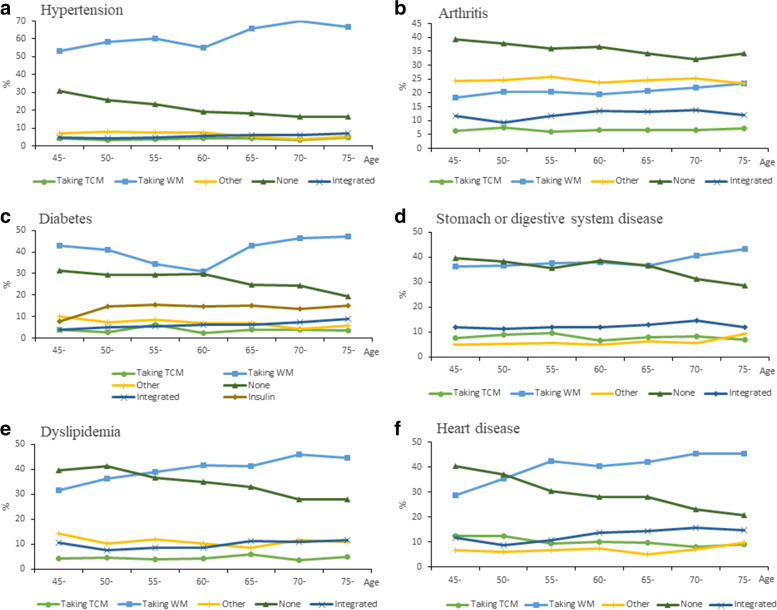
Prevalence of medical choices by age

According to age distribution, the medical choices of hypertension, arthritis, diabetes, stomach or digestive system diseases, dyslipidemia and heart disease patients are visualized. As shown in Fig. 1, the incidence of various medical choices differs among the six diseases. The proportion of patients who choose to take WM and those who do not take treatment is higher in all diseases and age groups. Among them, the proportion of arthritis patients in all age groups who choose not to be treated is higher than that of taking medicine. The proportion of patients under 50 with stomach disease, dyslipidemia, and heart disease who choose not to be treated is higher than that of taking medication. The proportion of patients who choose IM has an overall upward trend with aging. The proportion of patients who choose to take TCM is relatively low in all diseases and age groups. With the increase of age, the prevalence remains basically stable.

Table 4 and Table 5 show that patients with chronic diseases have the highest SRH score of 5 and the lowest of 1, and the highest depression score of 1, and the lowest of 0. From the average point of view, patients with heart disease have the highest SRH score of 3.433, which is poorer, while those with hypertension have better SRH. Patients with arthritis are more likely to have depression, and patients with hypertension show relatively less depression in the ten problems. The overall subjective health level of middle-aged and elderly people in the sample is between “average” and “poor”, and the overall depression is “mild depression”.

**Table 4:** Comparison of SRH of patients with six chronic diseases

	Min Value	Max Value	Mean	Standard Deviation	Obs
SRH					
Hypertension	1	5	3.201	0.977	6685
Arthritis	1	5	3.291	0.954	6729
Diabetes	1	5	3.384	0.926	2194
Stomach disease	1	5	3.287	0.954	5313
Dyslipidemia	1	5	3.282	0.967	3802
Heart disease	1	5	3.433	0.945	3409

**Table 5:** Comparison of depression of patients with six chronic diseases

	Min Value	Max Value	Mean	Standard Deviation	Obs
Depression					
Hypertension	0	1	0.366	0.482	5726
Arthritis	0	1	0.444	0.497	5739
Diabetes	0	1	0.386	0.487	1921
Stomach disease	0	1	0.436	0.496	4618
Dyslipidemia	0	1	0.374	0.484	3371
Heart disease	0	1	0.428	0.495	2974

### Association between medical choices and SRH of patients with six chronic diseases

Table 6 presents the estimated results of the association between different medical choices and SRH of patients with chronic diseases based on the ordered Logit model. Specifically: Taking TCM is significantly positively associated with the SRH results of patients with chronic diseases other than hypertension. Both taking WM and taking IM have significant positive correlations with SRH results. Treatment methods other than medication are significantly and positively associated with the SRH of patients except diabetes. And through the results of the marginal effect, it can be seen that under the condition that other influencing factors remain unchanged, taking TCM will increase the probability that the SRH score of patients with heart disease, arthritis, stomach disease, diabetes and dyslipidemia becomes 5 (that is, the SRH is very poor) by 4.4%, 1.6%, 3.9%, 3.7%, and 4.2%, respectively. Taking WM will increase the probability that the SRH score of patients with heart disease, hypertension, arthritis, stomach disease, diabetes and dyslipidemia becomes5 by 6.2%, 2.7%, 3.9%, 4.2%, 5.4%, and 5.7%, respectively. Taking IM will increase the probability that the SRH score of patients with six diseases becomes 5 by 11%, 6.2%, 8.1%, 7.2%, 6.8%, and 9.5%, respectively. Patients with chronic diseases who take IM have a higher probability of self-rated health deterioration. To a large extent, it shows that taking medication or other treatments are related to worse SRH compared with the choice of no treatment, which is related to the development trend and stage of chronic diseases, and may also be determined by the objective physiological conditions and subjective mentality of patients with chronic diseases. Patients with early chronic diseases generally do not have particularly adverse symptoms, while patients with more severe conditions often need to adhere to medication, pay a greater price for treatment, and feel poorer about themselves.

**Table 6:** Regression results of the association between medical choice and SRH

VARIABLES	Heart disease		Hypertension		Arthritis		Stomach disease		Diabetes		Dyslipidemia	
	coefficient	Marginal effect	coefficient	Marginal effect	coefficient	Marginal effect	coefficient	Marginal effect	coefficient	Marginal effect	coefficient	Marginal effect
Taking Chinese traditional medicine	0.539^***^	0.044^***^	0.062	0.004	0.223^**^	0.016^**^	0.530^***^	0.039^***^	0.464^**^	0.037^*^	0.564^***^	0.042^***^
	**(0.117)**	**(0.011)**	(0.126)	(0.008)	**(0.097)**	**(0.007)**	**(0.103)**	**(0.009)**	**(0.218)**	**(0.020)**	**(0.151)**	**(0.014)**
Taking Western modern medicine	0.708^***^	0.062^***^	0.399^***^	0.027^***^	0.496^***^	0.039^***^	0.562^***^	0.042^***^	0.632^***^	0.054^***^	0.708^***^	0.057^***^
	**(0.080)**	**(0.007)**	**(0.059)**	**(0.004)**	**(0.064)**	**(0.006)**	**(0.062)**	**(0.005)**	**(0.096)**	**(0.008)**	**(0.073)**	**(0.006)**
Other treatments	0.785^***^	0.071^***^	0.257^**^	0.016^**^	0.411^***^	0.031^***^	0.406^***^	0.029^***^	0.188	0.013	0.371^***^	0.026^***^
	**(0.136)**	**(0.015)**	**(0.105)**	**(0.007)**	**(0.060)**	**(0.005)**	**(0.117)**	**(0.009)**	(0.173)	(0.013)	**(0.107)**	**(0.008)**
Combination of Traditional Chinese and Western Medicine	1.082^***^	0.110^***^	0.781^***^	0.062^***^	0.874^***^	0.081^***^	0.850^***^	0.072^***^	0.763^***^	0.068^***^	1.044^***^	0.095^***^
	**(0.109)**	**(0.013)**	**(0.109)**	**(0.011)**	**(0.077)**	**(0.009)**	**(0.087)**	**(0.009)**	**(0.179)**	**(0.019)**	**(0.112)**	**(0.013)**
Gender	-0.292^***^	-0.030^***^	0.005	0.000	-0.077	-0.007	0.002	0.000	0.075	0.007	-0.073	-0.006
	(0.076)	(0.008)	(0.055)	(0.004)	(0.054)	(0.005)	(0.061)	(0.005)	(0.097)	(0.009)	(0.073)	(0.006)
Age	0.009^**^	0.001^**^	0.011^***^	0.001^***^	0.015^***^	0.001^***^	0.021^***^	0.002^***^	0.017^***^	0.002^***^	0.017^***^	0.001^***^
	(0.004)	(0.000)	(0.003)	(0.000)	(0.003)	(0.000)	(0.003)	(0.000)	(0.005)	(0.000)	(0.004)	(0.000)
Income	-0.048^***^	-0.005^***^	-0.032^***^	-0.002^***^	-0.032^***^	-0.003^***^	-0.089^***^	-0.002^***^	-0.062^***^	-0.006^***^	-0.034^***^	-0.003^***^
	(0.010)	(0.001)	(0.007)	(0.001)	(0.006)	(0.001)	(0.016)	(0.001)	(0.012)	(0.001)	(0.009)	(0.001)
Education	-0.092^***^	-0.009^***^	-0.064^***^	-0.005^***^	-0.075^***^	-0.006^***^	-0.0221^***^	-0.007^***^	-0.091^***^	-0.008^***^	-0.096^***^	-0.008^***^
	(0.019)	(0.002)	(0.014)	(0.001)	(0.015)	(0.001)	(0.007)	(0.001)	(0.023)	(0.002)	(0.018)	(0.002)
Social Activities	-0.311^***^	-0.032^***^	-0.240^***^	-0.018^***^	-0.205^***^	-0.018^***^	-0.217^***^	-0.018^***^	-0.335^***^	-0.031^***^	-0.381^***^	-0.034^***^
	(0.067)	(0.007)	(0.048)	(0.004)	(0.047)	(0.004)	(0.054)	(0.005)	(0.084)	(0.008)	(0.065)	(0.006)
Drinking	0.234^***^	0.024^***^	0.252^***^	0.019^***^	0.188^***^	0.016^***^	0.236^***^	0.020^***^	0.220^***^	0.021^***^	0.301^***^	0.027^***^
	(0.045)	(0.005)	(0.031)	(0.002)	(0.031)	(0.003)	(0.035)	(0.003)	(0.056)	(0.005)	(0.042)	(0.004)
Region (Central Region)	0.250^***^	0.026^***^	0.392^***^	0.028^***^	0.313^***^	0.026^***^	0.220^***^	0.017^***^	0.366^***^	0.032^***^	0.275^***^	0.023^***^
	(0.087)	(0.009)	(0.060)	(0.004)	(0.064)	(0.005)	(0.069)	(0.005)	(0.101)	(0.009)	(0.078)	(0.007)
Region (Western Region)	0.120	0.012	0.389^***^	0.027^***^	0.256^***^	0.020^***^	0.314^***^	0.025^***^	0.394^***^	0.035^***^	0.277^***^	0.023^***^
	(0.084)	(0.008)	(0.058)	(0.004)	(0.060)	(0.005)	(0.067)	(0.005)	(0.104)	(0.009)	(0.079)	(0.007)
Region (Northeast Region)	0.023	0.002	0.362^***^	0.025^***^	0.300^***^	0.024^***^	0.322^***^	0.026^**^	0.551^***^	0.052^***^	0.379^***^	0.033^***^
	(0.109)	(0.010)	(0.096)	(0.007)	(0.105)	(0.009)	(0.121)	(0.011)	(0.166)	(0.018)	(0.123)	(0.012)
Medical insurance	0.055^*^	0.006^*^	0.050^**^	0.004^**^	0.068^***^	0.006^***^	0.068^***^	0.006^***^	0.038	0.004	0.066^**^	0.006^**^
	(0.029)	(0.003)	(0.021)	(0.002)	(0.023)	(0.002)	(0.025)	(0.002)	(0.035)	(0.003)	(0.026)	(0.002)
Observations	3,409		6,685		6,729		5,313		2,194		3,802	

Due to space limitations, the marginal effect prediction coefficient and standard error are displayed with SRH of 5 (very poor). Significance level: *p < 0.1, **p < 0.05, ***p < 0.01

Regarding control variables, regardless of regression coefficients or marginal effects, variables such as age, personal income, education, social activities, drinking, region, and medical insurance will all have significant association with patients’ SRH. The regression results show that the older the patient is, the more likely that SRH will deteriorate. With the increase of the patient’s income and education level, both the anti-risk ability of personal wealth and health literacy will improve. The patient will pay more attention to health care, and naturally give his SRH a higher score. Compared with non-social groups, patients who participate in social activities tend to have higher SRH. Non-drinking is related to worse SRH, which may reflect the protective effect of moderate drinking (more than once a month) on chronic diseases, similar to the results of some epidemiological studies [33]. Compared with the eastern region, the economic development of the central, western and northeastern regions lags behind. Due to the relative decline in the ability to protect the health of residents, patients in these regions have poor SRH.

### Association between medical choices and depression of patients with six chronic diseases

Table 7 presents the estimated results of the association between different medical choices and depression of patients with chronic diseases based on the Logit model. Taking TCM is related to depression in patients with heart disease and stomach problems. Taking WM and IM is related to depression in patients with five other chronic diseases except diabetes. Depression in patients with arthritis is related to treatment other than medication, and depression in patients with diabetes is related to insulin injections.

**Table 7:** Regression results of the association between medical choice and depression

VARIABLES	Heart disease		Hypertension		Arthritis		Stomach disease		Diabetes		Dyslipidemia	
	coefficient	Marginal effect	coefficient	Marginal effect	coefficient	Marginal effect	coefficient	Marginal effect	coefficient	Marginal effect	coefficient	Marginal effect
Taking Chinese traditional medicine	0.274^*^	0.060^*^	0.054	0.011	0.156	0.036	0.286^**^	0.065^**^	0.002	0.000	0.125	0.026
	**(0.143)**	**(0.032)**	(0.156)	(0.033)	(0.113)	(0.026)	**(0.120)**	**(0.027)**	(0.278)	(0.059)	(0.187)	(0.039)
Taking Western modern medicine	0.445^***^	0.099^***^	0.199^***^	0.042^***^	0.378^***^	0.088^***^	0.381^***^	0.086^***^	0.047	0.010	0.474^***^	0.102^***^
	**(0.095)**	**(0.021)**	**(0.075)**	**(0.016)**	**(0.077)**	**(0.018)**	**(0.073)**	**(0.017)**	(0.117)	(0.025)	**(0.088)**	**(0.019)**
Other treatments	-0.064	-0.014	-0.198	-0.040	0.150^**^	0.035^**^	0.126	0.028	-0.140	-0.029	0.011	0.002
	(0.173)	(0.037)	(0.134)	0.027	**(0.072)**	**(0.017)**	(0.142)	(0.032)	(0.216)	(0.045)	(0.135)	(0.027)
Combination of Traditional Chinese and Western Medicine	0.617^***^	0.139^***^	0.393^***^	0.085^***^	0.623^***^	0.146^***^	0.725^***^	0.166^***^	-0.088	-0.018	0.599^***^	0.130^***^
	**(0.130)**	**(0.029)**	**(0.138)**	**(0.030)**	**(0.093)**	**(0.022)**	**(0.104)**	**(0.024)**	(0.225)	(0.047)	**(0.134)**	**(0.030)**
Gender	0.338^***^	0.075^***^	0.432^***^	0.092^***^	0.318^***^	0.073^***^	0.400^***^	0.090^***^	0.597^***^	0.126^***^	0.434^***^	0.092^***^
	(0.093)	(0.020)	(0.068)	(0.014)	(0.065)	(0.015)	(0.073)	(0.016)	(0.118)	(0.024)	(0.089)	(0.019)
Age	0.001	0.000	0.000	0.000	0.005	0.001	0.013^***^	0.003^***^	0.015^**^	0.003^**^	0.007	0.002
	(0.005)	(0.001)	(0.003)	(0.001)	(0.003)	(0.001)	(0.004)	(0.001)	(0.006)	(0.001)	(0.005)	(0.001)
Income	-0.030^***^	-0.007^***^	-0.029^***^	-0.006^***^	-0.037^***^	-0.008^***^	-0.126^***^	-0.010^***^	-0.048^***^	-0.010^***^	-0.047^***^	-0.010^***^
	(0.012)	(0.003)	(0.008)	(0.002)	(0.008)	(0.002)	(0.019)	(0.002)	(0.015)	(0.003)	(0.011)	(0.005)
Education	-0.149^***^	-0.033^***^	-0.139^***^	-0.030^***^	-0.115^***^	-0.027^***^	-0.043^***^	-0.028^***^	-0.154^***^	-0.033^***^	-0.112^***^	-0.024^***^
	(0.023)	0.005	(0.018)	(0.004)	(0.017)	(0.004)	(0.008)	(0.004)	(0.029)	(0.006)	(0.022)	(0.005)
Social Activities	-0.210^***^	-0.047^***^	-0.139^**^	-0.030^**^	-0.131^**^	-0.030^**^	-0.157^**^	-0.035^**^	-0.318^***^	-0.067^***^	-0.293^***^	-0.062^***^
	(0.081)	(0.018)	(0.059)	0.013	(0.056)	(0.013)	(0.064)	(0.014)	(0.102)	(0.021)	(0.079)	(0.017)
Drinking	0.030	0.007	0.053	0.011	0.042	0.010	0.087^**^	0.020^**^	-0.014	-0.003	0.094^*^	0.020^*^
	(0.055)	(0.012)	(0.039)	(0.008)	(0.037)	(0.008)	(0.042)	(0.009)	(0.070)	(0.015)	(0.052)	(0.011)
Region (Central Region)	0.385^***^	0.085^***^	0.473^***^	0.099^***^	0.444^***^	0.102^***^	0.369^***^	0.082^***^	0.410^***^	0.085^***^	0.360^***^	0.075^***^
	(0.109)	0.024	(0.076)	(0.016)	(0.078)	(0.018)	(0.085)	(0.019)	(0.127)	(0.026)	(0.098)	(0.020)
Region (Western Region)	0.562^***^	0.125^***^	0.637^***^	0.136^***^	0.471^***^	0.108^***^	0.458^***^	0.103^***^	0.686^***^	0.146^***^	0.516^***^	0.109^***^
	(0.103)	(0.023)	(0.074)	(0.016)	(0.073)	(0.017)	(0.081)	(0.018)	(0.130)	(0.027)	(0.099)	(0.021)
Region (Northeast Region)	0.289^**^	0.063^**^	0.302^***^	0.062^**^	0.258^**^	0.058^**^	0.214	0.047	0.518^**^	0.108^**^	0.328^**^	0.068^**^
	(0.131)	(0.029)	(0.117)	(0.024)	(0.124)	(0.028)	(0.142)	(0.032)	(0.200)	(0.043)	(0.146)	(0.031)
Medical insurance	0.134^***^	0.030^***^	0.121^***^	0.026^***^	0.104^***^	0.024^***^	0.072^**^	0.016^**^	0.092^**^	0.019^**^	0.081^**^	0.017^**^
	(0.035)	(0.008)	(0.027)	(0.006)	(0.028)	(0.006)	(0.031)	(0.007)	(0.044)	(0.009)	(0.033)	(0.007)
Observations	2,974		5,726		5,739		4,618		1,921		3,371	

Significance level: *p < 0.1, **p < 0.05, ***p < 0.01

From the results of marginal effects, it can be seen that under the condition that other influencing factors remain unchanged, taking TCM will increase the probability of depression in patients with heart disease and stomach disease by 6.0% and 6.5% respectively, while taking WM will increase the probability by 9.9% and 8.6% respectively, which is higher than that of taking TCM. Taking IM will increase the probability of depression in patients with heart disease, hypertension, arthritis, stomach disease and dyslipidemia by 13.9%, 8.5%, 14.6%, 16.6%, and 13.0%, respectively. Patients with chronic diseases who take IM have a higher probability of suffering from depression. This is mainly because patients are usually in the middle and late stages of the development of chronic diseases when taking Chinese medicine and Western medicine at the same time. As the symptoms intensify and the course of the disease continues to develop, the patient will be more prone to depression and negative emotions. In terms of control variables, female, low income, low education, no social activities, living in the central and western regions, and medical insurance variable are also significantly related to depression.

## Discussion

In this sample of Chinese patients with six chronic diseases, we use the ordered Logit regression model and the Logit regression model to study the association between medical choices and patients’ SRH and depression. And by including six common chronic diseases, the medical choices and health effects of patients with different diseases are compared.

### Medical treatment choices of patients

On the whole, patients with different chronic diseases have different medical choices, but they all rely on taking WM. The highest prevalence of taking TCM alone is 10.03% (95%CI 9.10% to 11.04%). This ratio is lower than the result of the previous study: the prevalence of TCM in chronic disease patients was more than 20% [28]. This is because this study differentiates between patients who take TCM alone and those who take combined TCM and WM, thus reducing the prevalence of taking TCM. At the same time, as the CHARLS follow-up survey is carried out year by year, the decline in the use of TCM for chronic diseases may also be related to the aggravation of the same group of patients with chronic diseases. In terms of specific diseases, the prevalence of taking western medicine in hypertensive patients is the highest among the six diseases. This is because Western medicine has reliable clinical evidence for blood pressure control [34], and it may also be related to patients’ perception of hypertension and beliefs in the Western medicine used [35]. The prevalence of arthritis patients choosing other treatment methods is the highest among the six diseases, and the prevalence of choosing IM is also relatively high. It shows that while western medicine is used to relieve the condition, traditional Chinese medicine has also played its unique regulating effect on different types of arthritis, such as dampness arthralgia, wandering arthritis and pyretic arthralgia [36]. The prevalence of diabetic patients taking TCM is the lowest among the six diseases, indicating that TCM is not suitable for long-term use as the main medication for hypoglycemic therapy. The prevalence of choosing TCM and IM for patients with stomach or digestive system diseases is relatively high. It can be seen that chronic gastric disease fits well with the “health conditioning” treatment of traditional Chinese medicine [37]. The prevalence of heart disease patients taking TCM is the highest among the six diseases. Based on the clinical efficacy of Chinese patent medicine for promoting blood circulation to treat coronary heart disease [38], patients can achieve the purpose of replenishing qi, promoting blood circulation and removing blood stasis by taking TCM.

### The association between medical treatment choices and health of patients with chronic diseases

By investigating the association between medical choices and health of patients with chronic diseases, we find that there is a certain difference between the physical health represented by SRH and the mental health represented by depression. Taking the non-treatment group as reference, in the SRH section, taking TCM has significant positive association with the other five diseases except hypertension. Both taking WM and taking IM show a significant positive correlation with the SRH of patients with six chronic diseases. In general, patients who take IM have a higher probability of self-rated health deterioration, and the probability of SRH worsened by taking WM is higher than that of taking TCM. Previous studies have shown that TCM has significant effects on chronic diseases such as hypertension [39] and arthritis [40]. However, some scholars believe that some test reports lack high-level evidence [41]. Compared with the results of previous studies, this study believes that taking TCM is the choice of patients with chronic diseases for early prevention and adjuvant treatment in the middle and late stages. “Treatment of disease before disease” is an important part of traditional Chinese medicine, realizing the unique role and advantages of TCM in disease prevention or early treatment [42]. Therefore, many patients use TCM for early prevention or treatment of chronic diseases [43]. This is consistent with the results of this study that taking TCM has significant association with the SRH of patients with chronic diseases but is less effective than taking WM. From the results of marginal effects, it can be seen that the medical choice type of IM has the most palpable association with the SRH of patients with chronic diseases. That is, combined medication is often associated with worse SRH. This is because the treatment of IM is usually used to improve the quality of late-stage diagnosis and treatment of chronic diseases to help patients relieve pain and improve health-related quality of life. The rapid aging of the Chinese population and the significant role of integrated traditional Chinese and western medicine mean that the demand for IM will be greater in the future, which has prompted China to systematically study and expand the theory and practice of IM [44], carry out the construction of integrated traditional Chinese and western medicine specialties for chronic diseases, formulate personalized chronic disease health management, and maintain the physical and mental health of chronically ill patients with long-term illnesses. At the same time, with the increasing popularity of TCM as a supplementary therapy of WM in various types of chronic diseases [25], adverse drug reactions caused by herbal-drug interactions have always been concerned [45]. Previous studies have found that many patients did not actively disclose the use of supplementary and alternative medicines to their healthcare professionals. Therefore, the formulation of clinical guidelines should pay attention to the use of supplementary drugs by their patients to ensure the rational use of Chinese and Western medicines and promote patient compliance with drugs [46]. In the section of depression, taking TCM is related to depression in patients with heart disease and stomach problems. Taking WM and IM makes patients with five chronic diseases more likely to be depressed except for diabetes. Depression in patients with arthritis is related to other treatments. Injecting insulin will affect the depression of diabetic patients. This result fully reflects the difference in disease types, and also shows that the association between taking TCM and patients’ depression is generally slighter than that of taking WM. The reason is that patients taking TCM should be mildly ill and have relatively high health-related quality of life. In addition, this may be related to the trust of older Chinese patients in Chinese medicine supported by traditional values [47]. Patients believe that different from the toxicity of chemical drugs, traditional Chinese medicine is a natural product with lower side effects and lighter psychological burden of taking the medicine.

### Diagnosed but untreated chronic disease patients

The large number of patients who have been diagnosed with chronic diseases but do not choose drugs or other treatments cannot be ignored. In this study, 20.27%, 35.56%, 26.62%, 35.53%, 34.50%, 29.09% of patients with hypertension, arthritis, diabetes, stomach disease, dyslipidemia, and heart disease did not take any treatment. Traditional health protection models (such as protection motivation theory) believe that health protection behaviors are a function of the probability and severity of health outcomes, the perceived effectiveness of protection behaviors, and the perceived cost and barriers to action (Weinstein 1993). Starting from the theory, combined with practical analysis, the reason why patients choose not to be treated may on the one hand be lack of health literacy [48]. They even hide their diseases with a taboo attitude and are unwilling to face treatment actively. On the other hand, it may be related to the expected titer [11]. When the patient’s physical condition is poor, and the perceived effectiveness of receiving treatment is average, coupled with the perceived unaffordable medical cost, it is possible to choose to give up treatment directly. Previous studies have shown that patients with lower education level and lower family income are more likely to refuse treatment because of their poor health and choose not to receive treatment after evaluating socio-economic factors and curative effects [49]. Earlier studies have also found that it is common for the elderly with advanced chronic diseases to refuse treatment. Treatment needs to balance benefits and burdens, and the refuse of treatment is also related to patients’ greater desire and understanding of prognostic information [50]. This suggests that medical staff should not only provide treatment for patients, but also communicate with patients appropriately, provide psychological counseling, and help patients increase their confidence in themselves. The reform of the medical system should focus on reducing the financial burden of patients and creating medical environment with diversified medical choices for patients.

### The trustworthiness of self-rated health

In this study, compared with the non-treatment group, each group of medical choices has negative impact on the SRH of patients with six diseases, which seems to be somewhat unexpected. This study mainly explains from the development stage of chronic diseases and patients’ subjective wishes, and does not rule out the bias of SRH evaluation indicator. Patients who choose not to be treated may just suffer from chronic diseases, so they don’t pay enough attention to the disease and feel good about themselves. They hope to reduce blood sugar, blood pressure and blood lipid and relieve stomach diseases by exercising and regulating diet. However, the elderly who take medicine or receive other treatments for a long time often come to the middle and late stages of chronic diseases, and can only maintain the status quo through taking medicine. Their overall health status is still poor, and psychologically they tend to discount their own health evaluations. SRH is one of the most commonly used indicators to measure health levels. It is not only widely used in cross-sectional data analysis, but also in tracking survey data analysis to measure changes in the health status of respondents over time. But it is undeniable that its subjectivity may make its accuracy questionable because its value is not only affected by the objective health of the respondents, but also by their evaluation standards. And empirical studies proved that there were significant differences in health evaluation standards among different groups of people [51]. In addition, through CFPS panel data research, Wu [52] found that in the population aged 45–70, the appearance of daily activity disorders would lead to a decline in health evaluation standards.

### Innovation

This research is somewhat innovative. When examining the health of patients with chronic diseases, our research pays attention to both disease types and medical choices for the first time. This can better reveal the prevalence of TCM and WM in several common chronic diseases and their association with the health of patients. For the measurement of health level, the inclusion of SRH representing physical health and depression representing mental health at the same time helps to understand the health level of patients with chronic diseases more comprehensively.

### Limitations

This study has some limitations in data. First of all, this is a cross-sectional study that can only introduce factors that affect the health of patients with chronic diseases, but cannot provide evidence of causality. Secondly, due to sample limitations, more patients with chronic diseases of other diseases cannot be included in the study. Third, the lack of detailed information on treatment methods hinders us from exploring different types of treatment situations.

## Conclusion

Through the analysis of research results, we have concluded that in the future, we can explore the control of chronic diseases or comorbidities from the perspective of integrated Chinese and Western medicine, but we need to pay attention to drug interactions and carry out safe combination drug research. Traditional Chinese medicine can expand the treatment choices for patients with chronic diseases, especially in the early stage of prevention. And compared with taking western medicine, there is no more correlation between taking traditional Chinese medicine and depression. The number of patients who have been diagnosed with chronic diseases but did not choose drugs or other treatment methods in this study should not be ignored, suggesting that policymakers may consider doing some work in reducing the economic burden of patients with chronic diseases and improving public health literacy.

## Supplementary Information

**Additional file 1.**

## Data Availability

Data is publicly available. See: https://charls.charlsdata.com/pages/Data/2018-charls-wave4/zh-cn.html

## Abbreviations

TCM: Traditional Chinese Medicine
SRH: Self-rated health
WM: Western medicine
IM: Integrated Chinese and Western medicine
