# Supplementary material for: Do medical treatment choices affect the health of chronic patients in middle and old age in China?—Evidence from CHARLS 2018
Source: BMC Public Health. 2022 May 10;22:937. doi: 10.1186/s12889-022-13309-3 (PMC9088154; doi:10.1186/s12889-022-13309-3)

***1 Independent and dependent variables selected in the questionnaire***

**1.1 Independent variable:**

**Medical treatment choice**


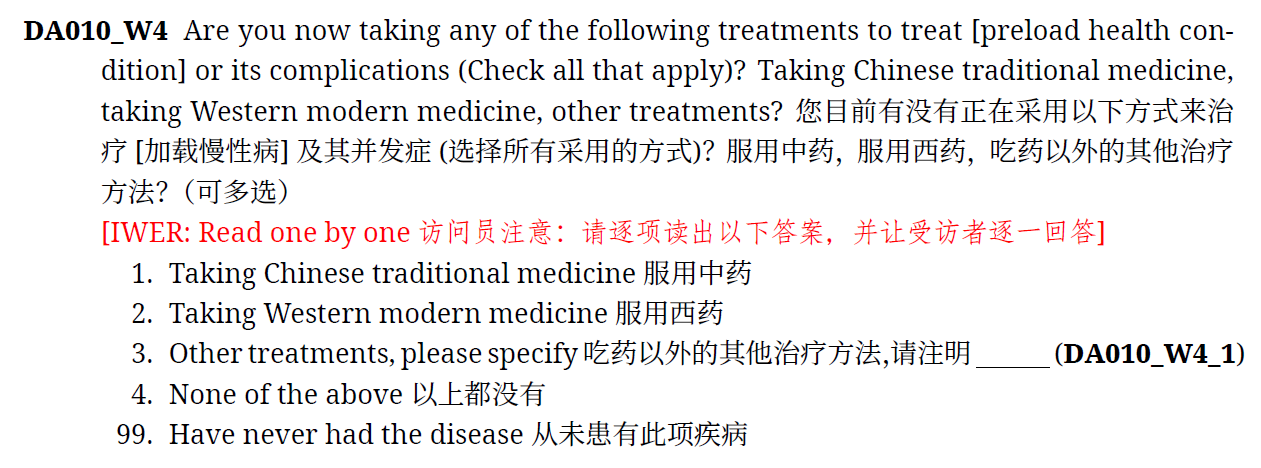


**1.2 Dependent variable:**

**Self-rated health**


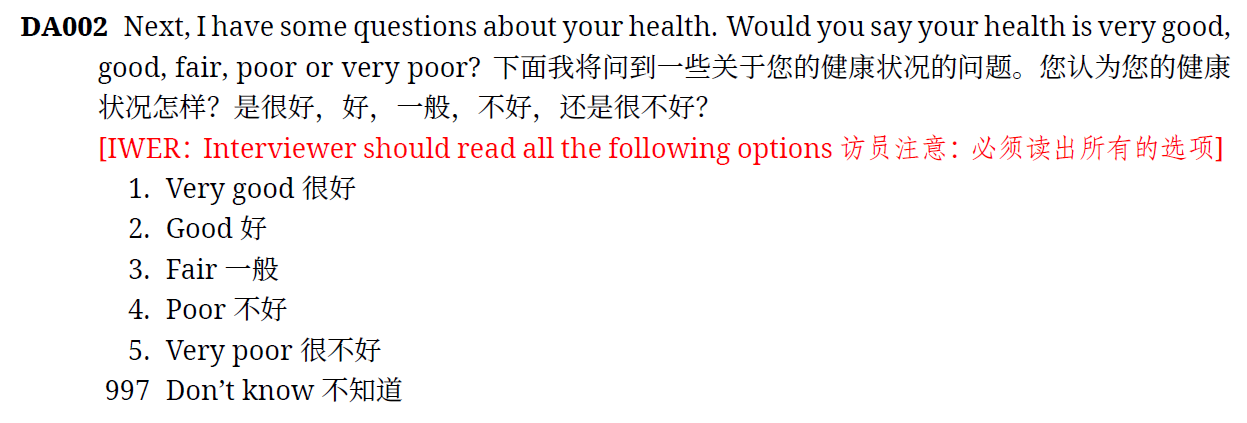


**Depression**
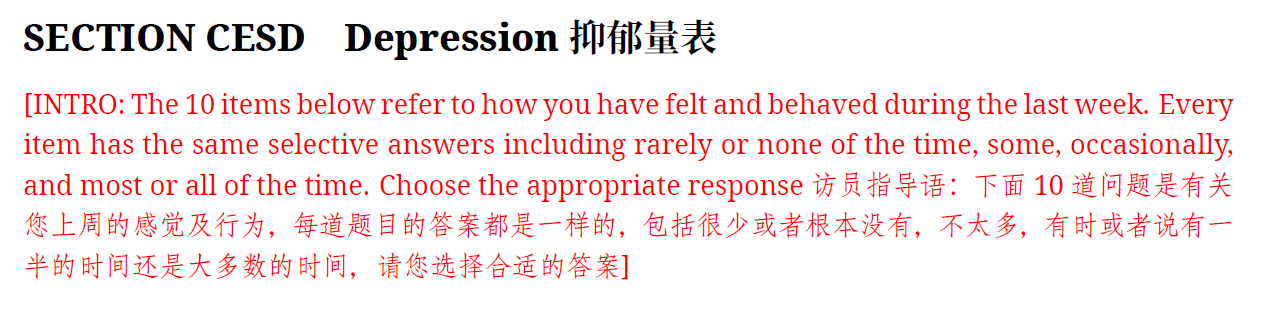


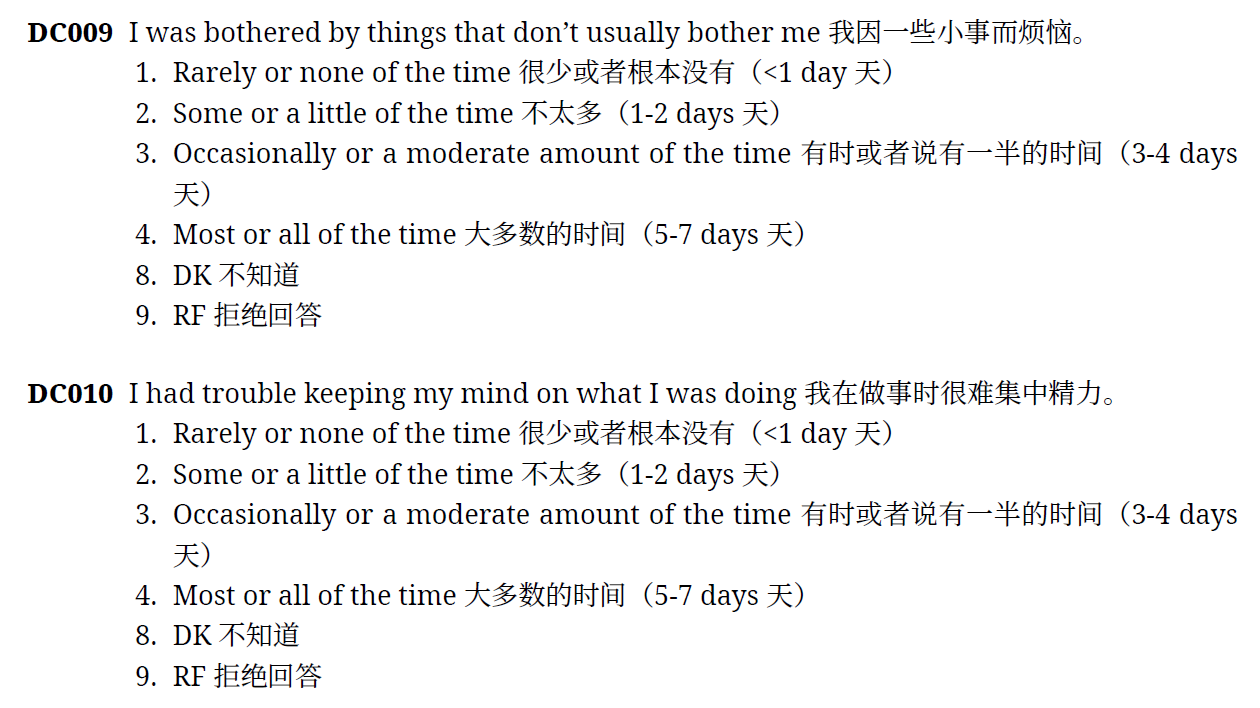


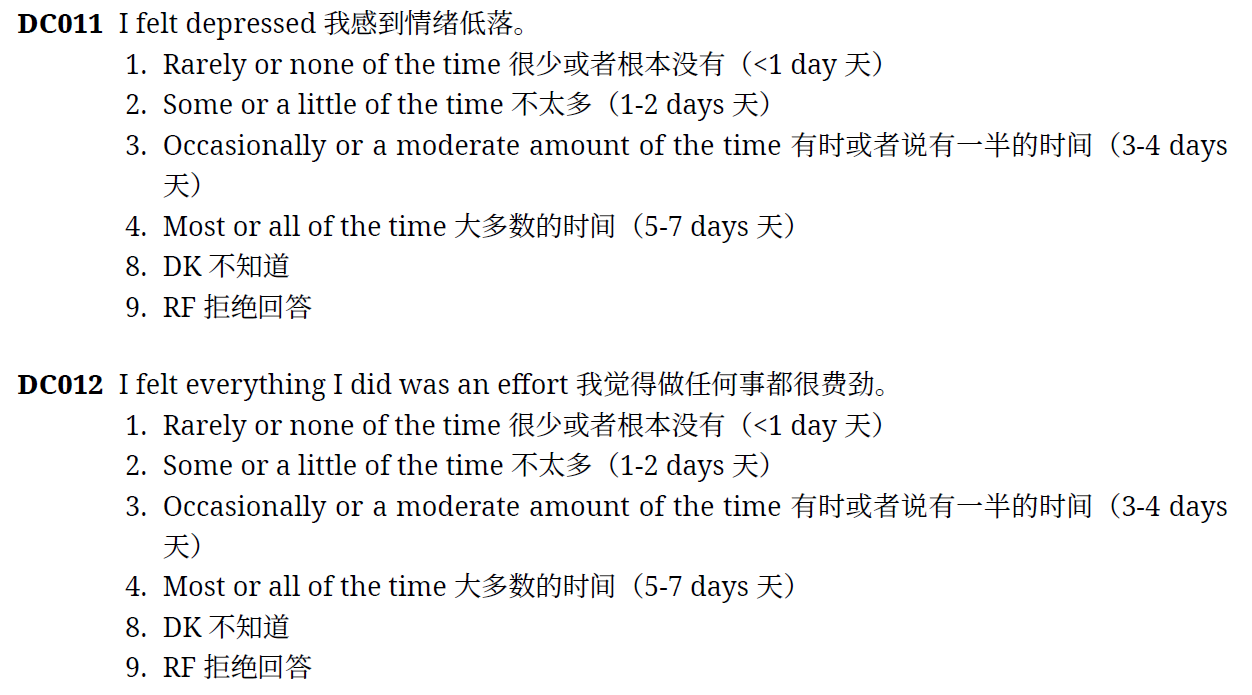


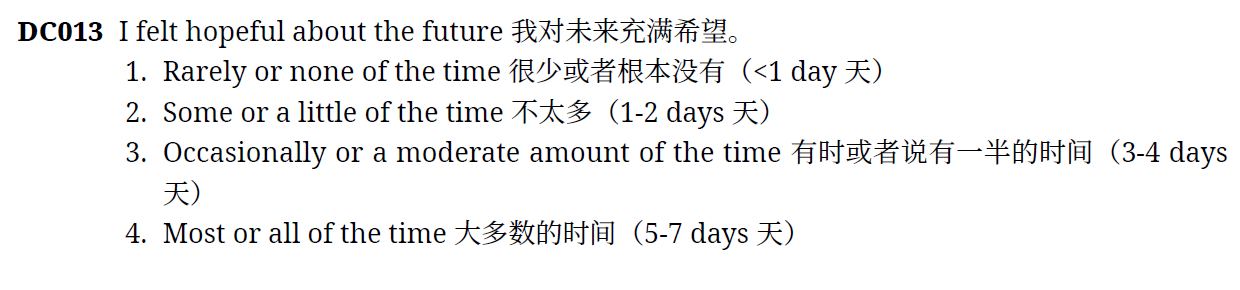


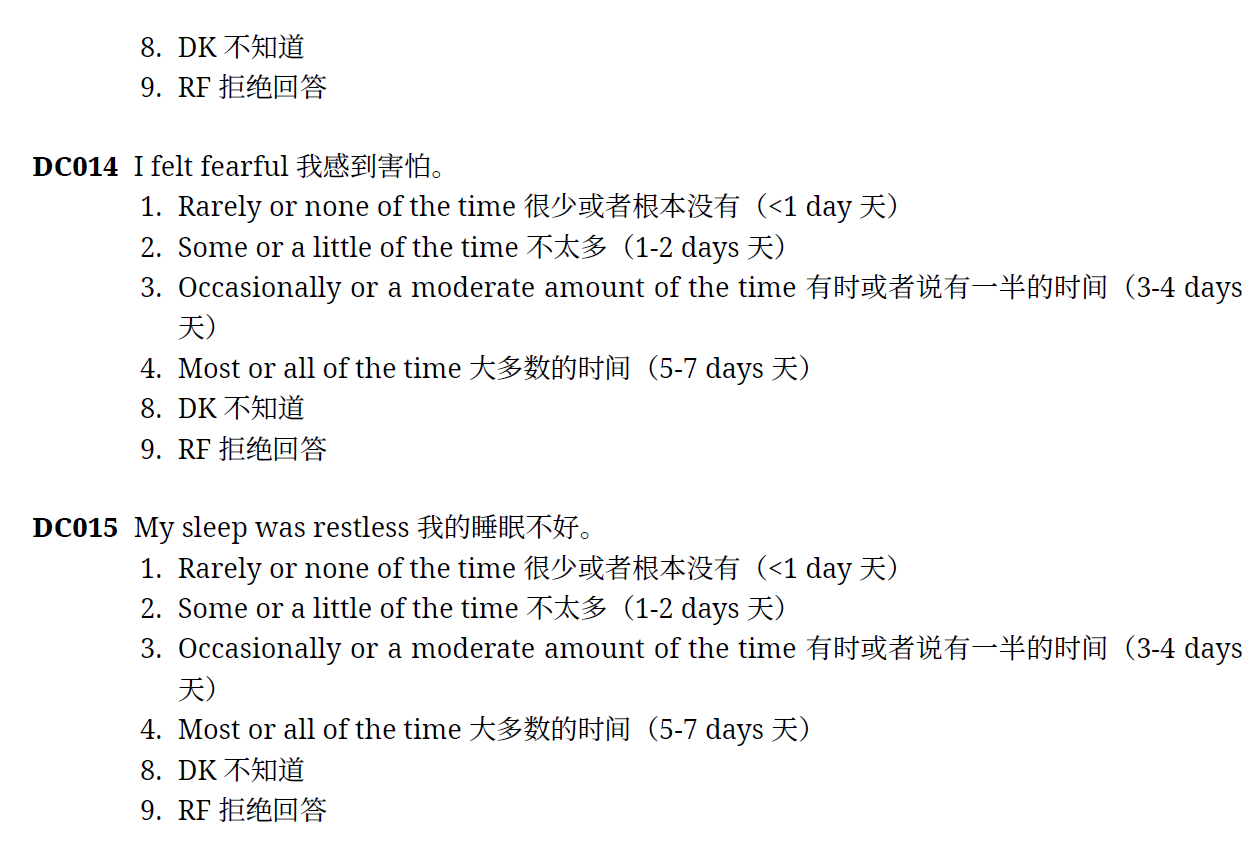


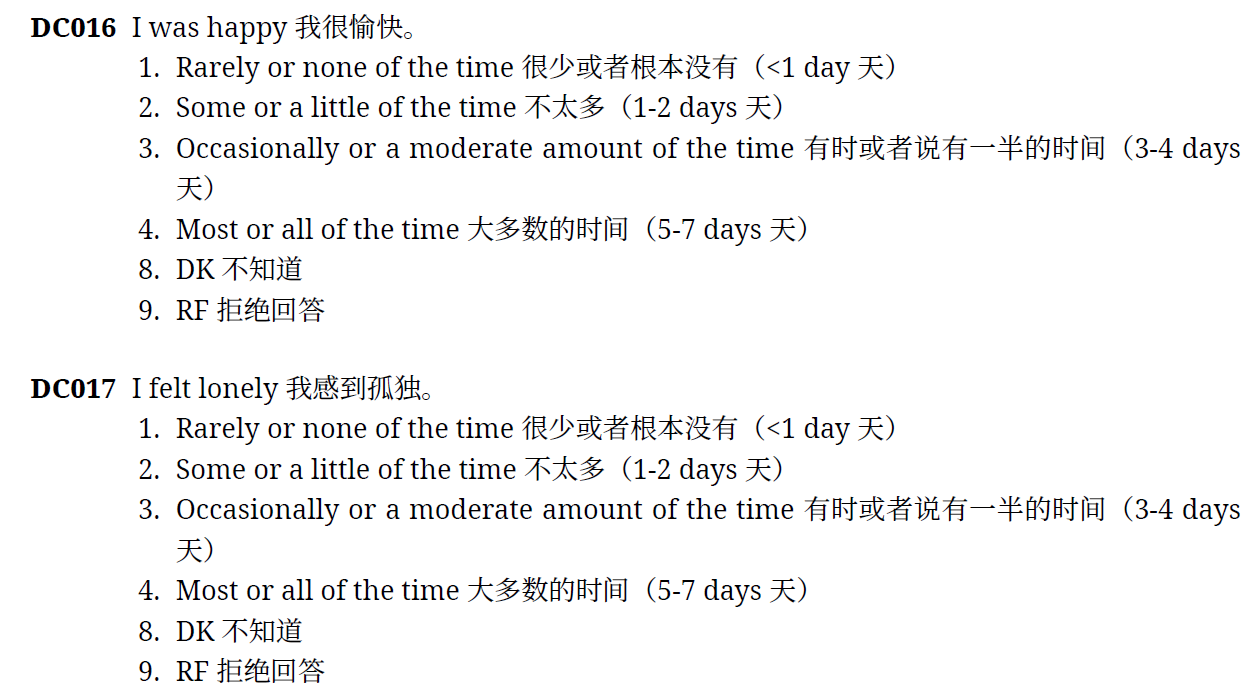


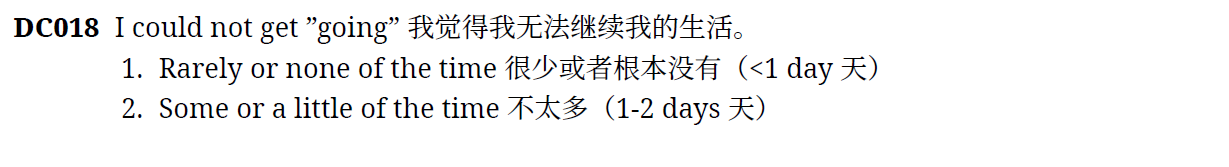


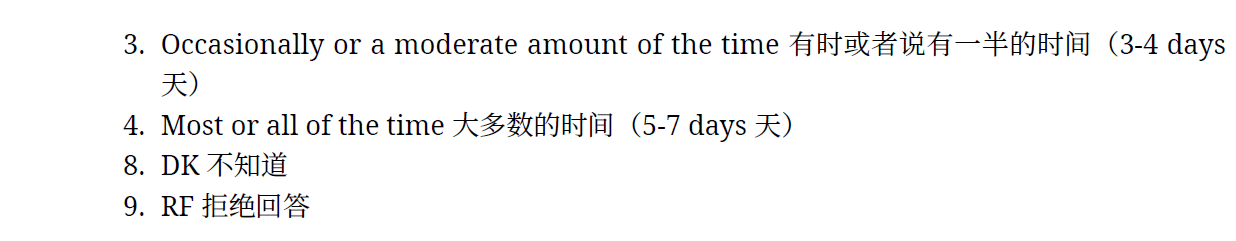


***2*** ***Chronic disease diagnosis***


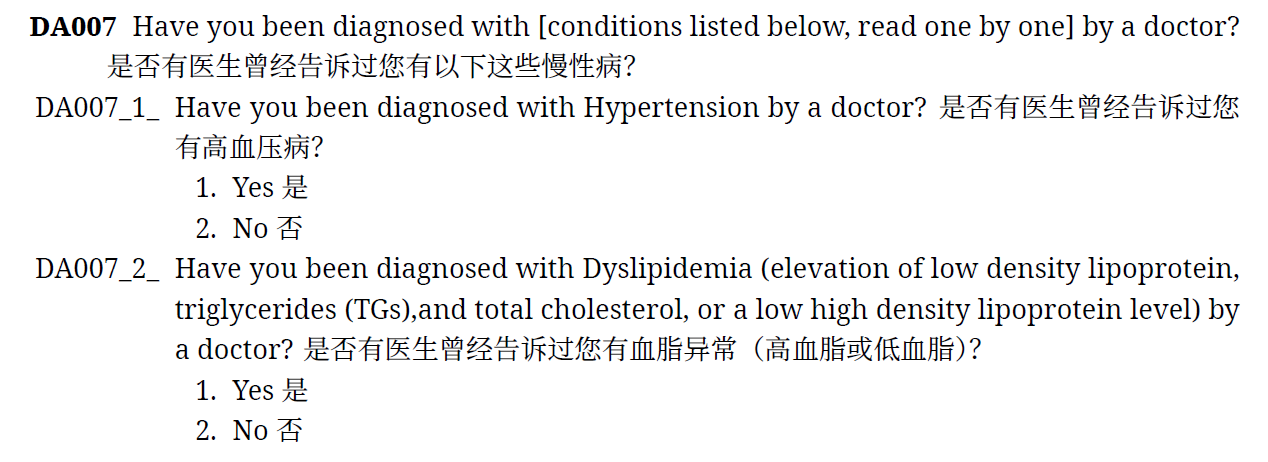


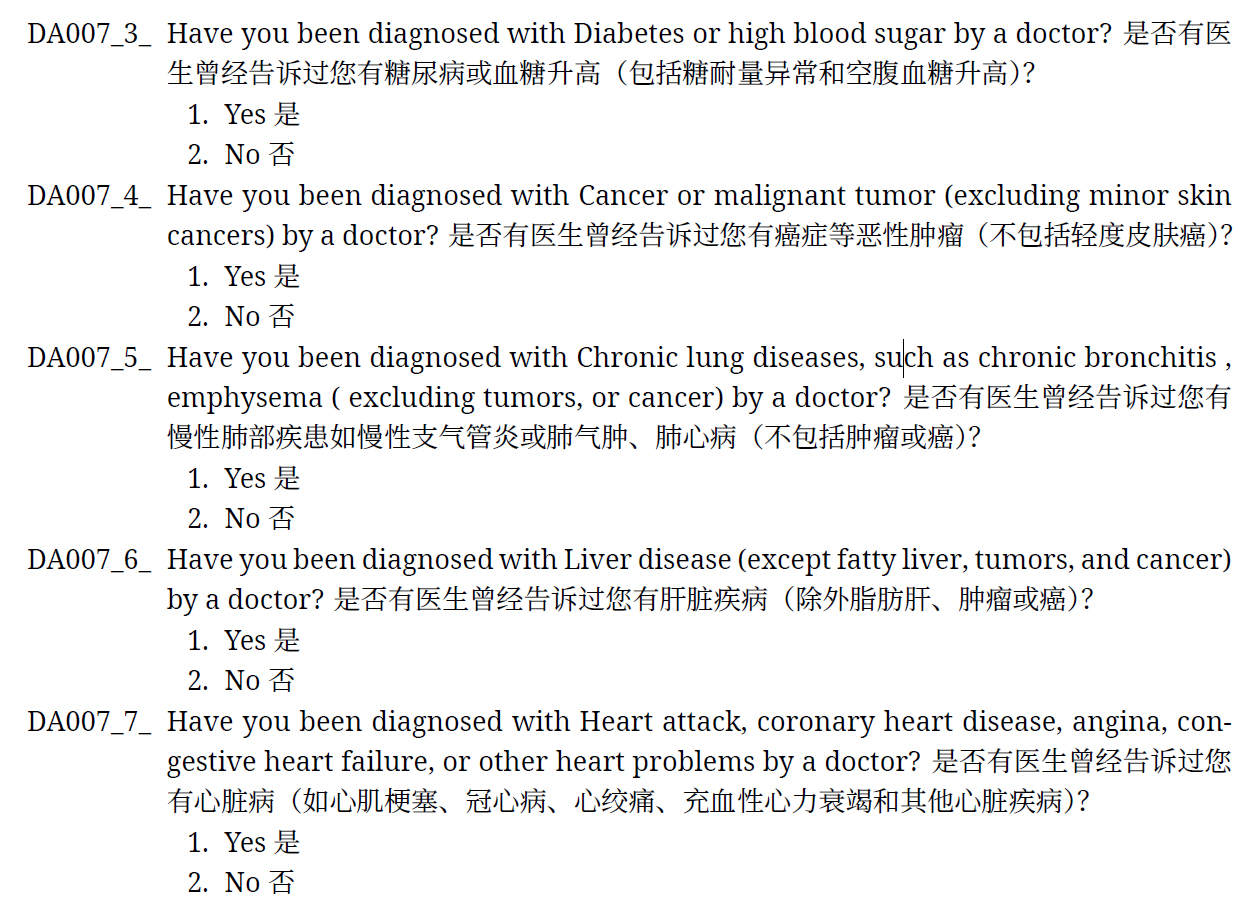


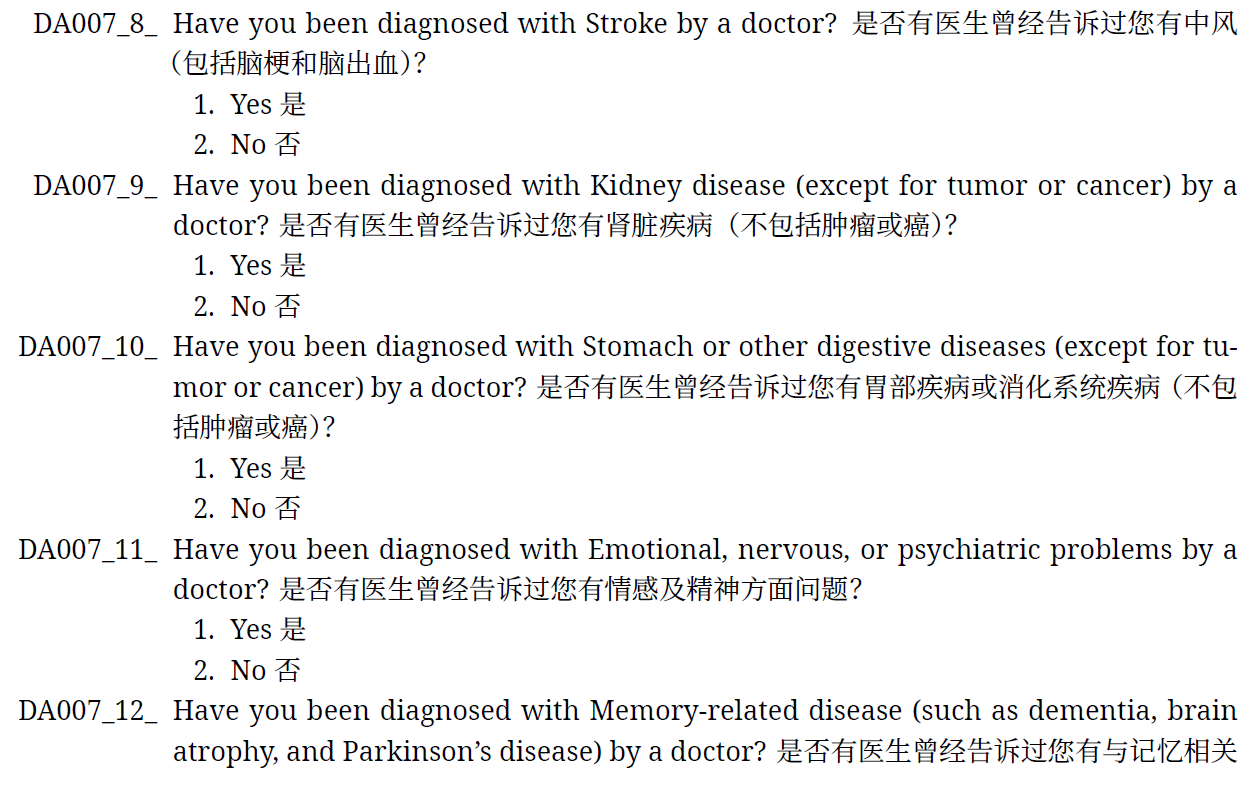


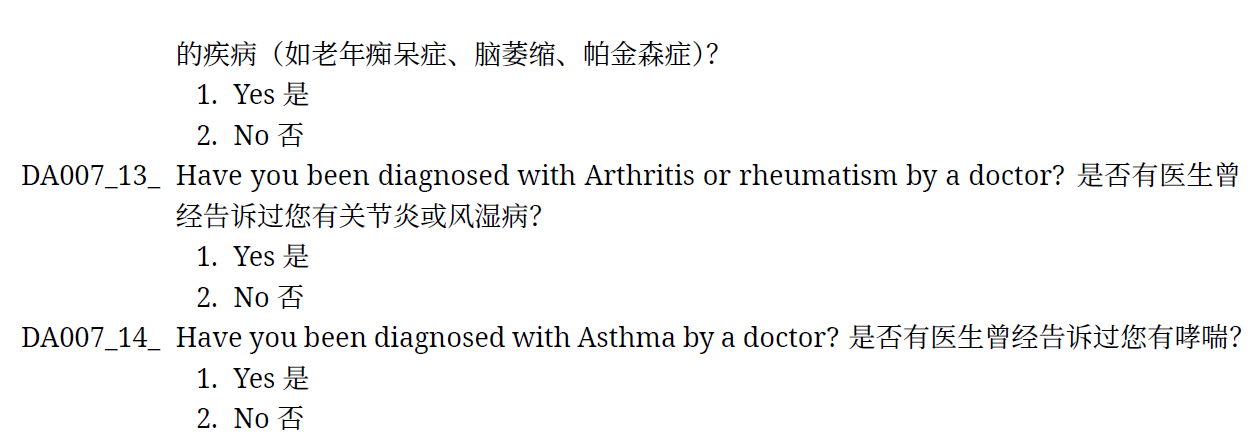


***3 Significance Test***

**Age** \
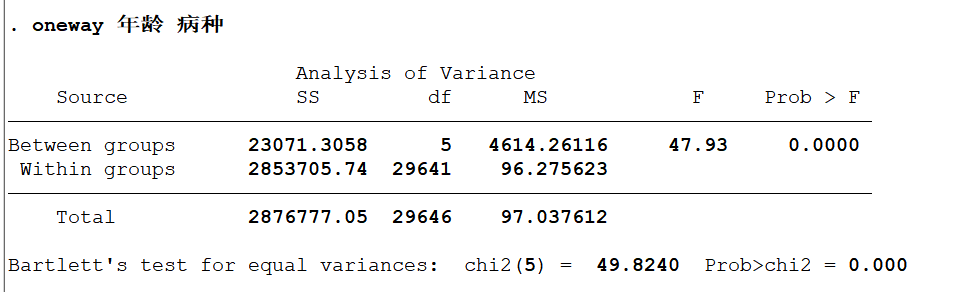


**Gender**


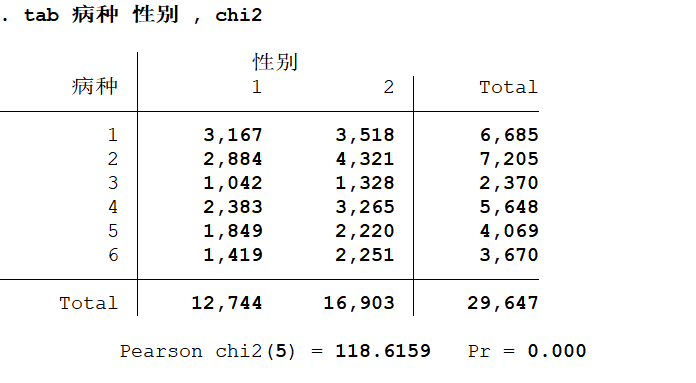


**Ln (Income)**


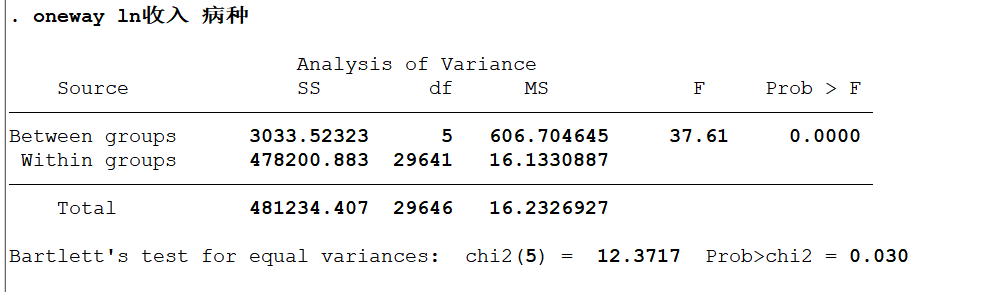


**Region**


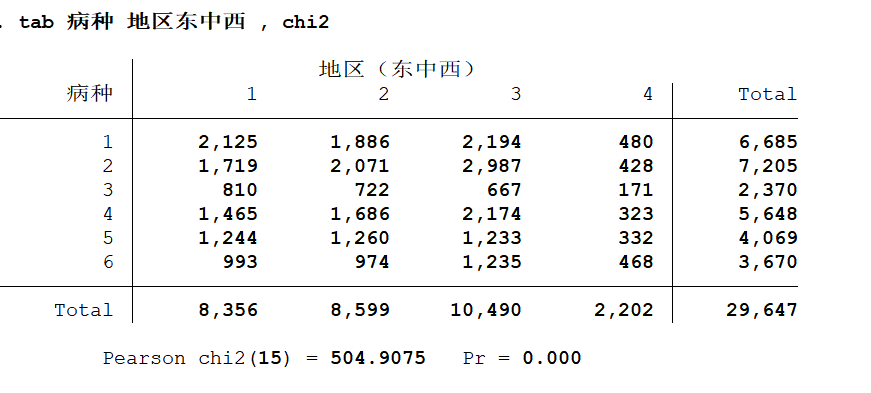


**Social activities**


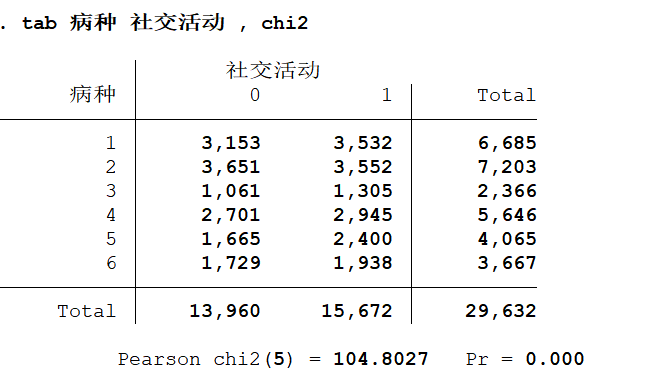


**Education**


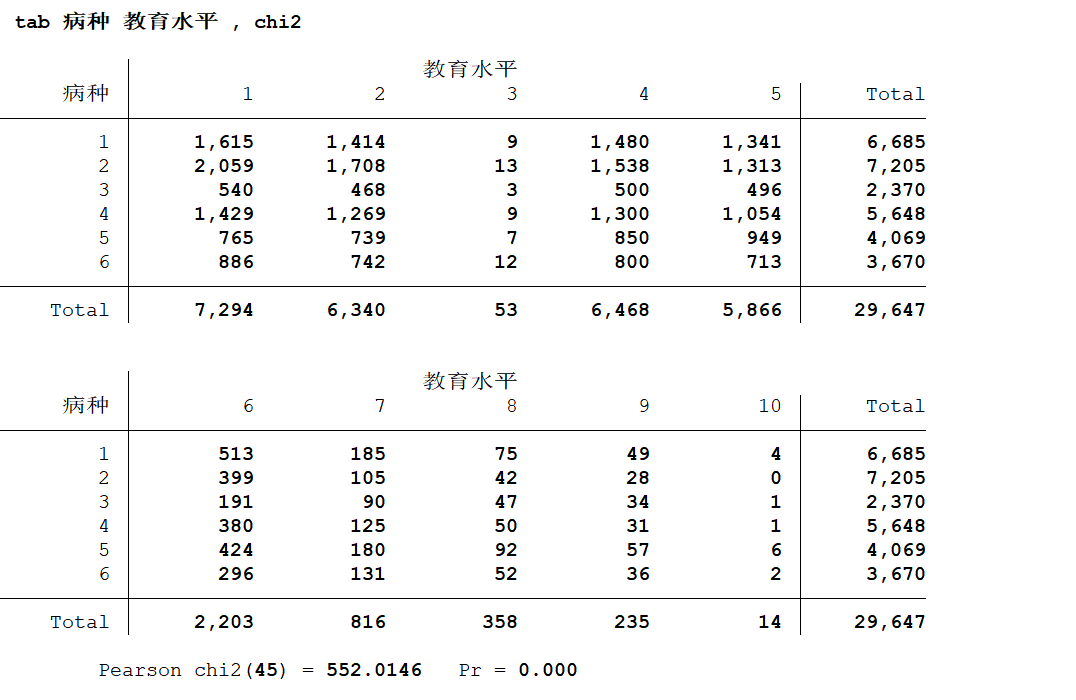


**Drinking**


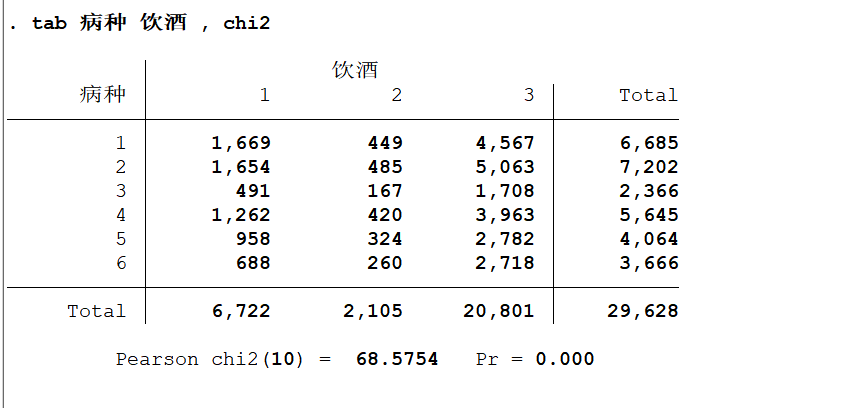


**Medical Insurance**


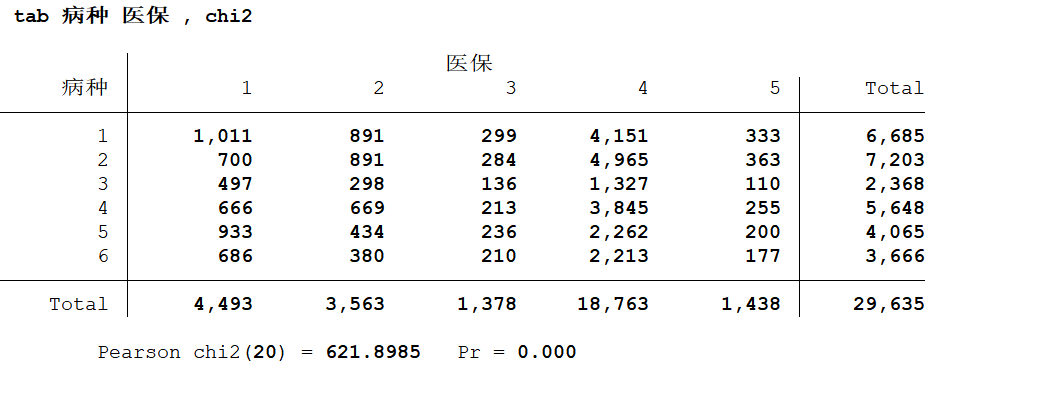


**Medical Treatment Choice**


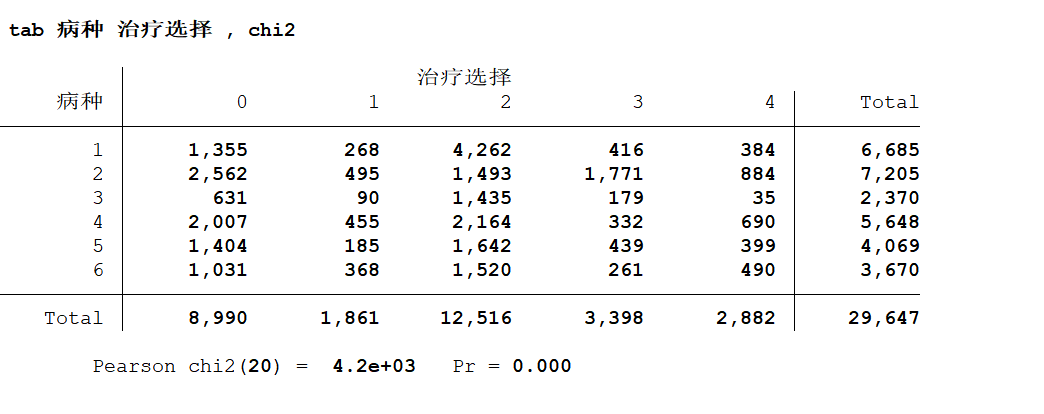

Supplement: Supplementary file 1 — Additional file 1. [file 12889_2022_13309_MOESM1_ESM.docx]
